# Usefulness of selective arterial calcium injection tests for functional pancreatic neuroendocrine tumors

**DOI:** 10.1038/s41598-020-80538-0

**Published:** 2021-01-08

**Authors:** Yutaka Nakano, Minoru Kitago, Masahiro Shinoda, Seishi Nakatsuka, Isao Kurihara, Hiroshi Yagi, Yuta Abe, Go Oshima, Shutaro Hori, Takahiro Yokose, Yuko Kitagawa

**Affiliations:** 1 Department of Surgery, Keio University School of Medicine, 35 Shinanomachi, Shinjuku-ku, Tokyo, 160-8582 Japan; 2 Department of Diagnostic Radiology, Keio University School of Medicine, 35 Shinanomachi, Shinjuku-ku, Tokyo, 160-8582 Japan; 3 Department of Nephrology, Endocrinology and Metabolism, Keio University School of Medicine, 35 Shinanomachi, Shinjuku-ku, Tokyo, 160-8582 Japan

**Keywords:** Gastrointestinal diseases, Imaging

## Abstract

The selective arterial calcium injection (SACI) test is useful for patients with functional pancreatic neuroendocrine tumors (F-PNETs). This study evaluated which patients with F-PNETs would benefit from the SACI test. We retrospectively analyzed the preoperative findings of patients on computed tomography (CT), magnetic resonance imaging (MRI), CT angiography (CTA), and the SACI test. Fourteen patients who underwent pancreatectomy between January 1997 and September 2016 for F-PNETs were evaluated. We classified these patients into groups A, B, and C; group A, one tumor detected by either CT or MRI; group B, multiple tumors detected; and group C, the tumor location was accordant on CT, MRI, and CTA, but the SACI test revealed another tumor. In group A, the tumor was also detected by CTA and the SACI test was positive on calcium injection. In group B, the focus tumor among the multiple tumors was detected by the SACI test. In group C, another tumor was identified by the SACI test, whose location was different from that detected using CT and MRI. The SACI test is more useful for multiple F-PNETs on CT or MRI. If CT or MRI detects a single tumor, the SACI test or CTA may be unnecessary.

## Introduction

The use of the selective arterial calcium injection (SACI) test has resulted in the increased performance of curative resection surgery for patients with functional pancreatic neuroendocrine tumors (F-PNETs), such as insulinomas and gastrinomas^1,2^. In 1991, Doppman et al.^2^ reported a method of localizing insulinomas using 0.025 mEq/kg calcium gluconate as the secretagogue. Moreover, the SACI test has a high diagnostic performance for the accurate localization of F-PNETs for curative resection when F-PNETs such as insulinomas and gastrinomas are usually small, solitary, and intrapancreatic^3–5^.

Other imaging studies, such as computed tomography (CT), magnetic resonance imaging (MRI), and CT angiography (CTA), can also be used to visualize endocrine tumors as hypervascular tumors surrounding pancreatoduodenal lesions. Although the specificity and sensitivity of these methods are both less than 70–80% for pancreatic tumors^6^, CT is the initial modality of choice to evaluate pancreatic lesions while MRI has a major role in pancreatic tumor characterization^7^. In contrast, compared to CT or MRI, the SACI test and CTA are relatively invasive examinations for patients with F-PNETs; therefore, these invasive examinations should not be performed if possible. Moreover, it is unclear which patients with F-PNETs would benefit from the SACI test, and only a few studies have determined the value of the SACI test compared to preoperative findings of CT, MRI, and CTA^5,8^. Therefore, in this study, we aimed to investigate the preoperative findings of the SACI test, CT, MRI, and CTA, and to determine the type of patients with F-PNETs in whom the SACI test would be very effective.

## Patients and methods

### Patients

Patients who underwent the SACI test and curative pancreatectomy for F-PNETs at our institution between January 1997 and September 2016 were retrospectively reviewed. All patients were histologically confirmed as having F-PNETs such as insulinomas or gastrinomas.

Along with the SACI test, all patients underwent other preoperative tumor localization procedures such as dynamic CT, dynamic MRI, and CTA. The results of the SACI test were compared with the findings on these procedures, as well as the surgical and pathological results.

We conducted this retrospective study using the “opt-out” method of our hospital. The study was approved by the Ethics Committee of Keio University School of Medicine, which waived the need of informed consent for our study, and conducted in accordance with the Helsinki Declaration of 1975.

### The SACI test

**Figure 1:**
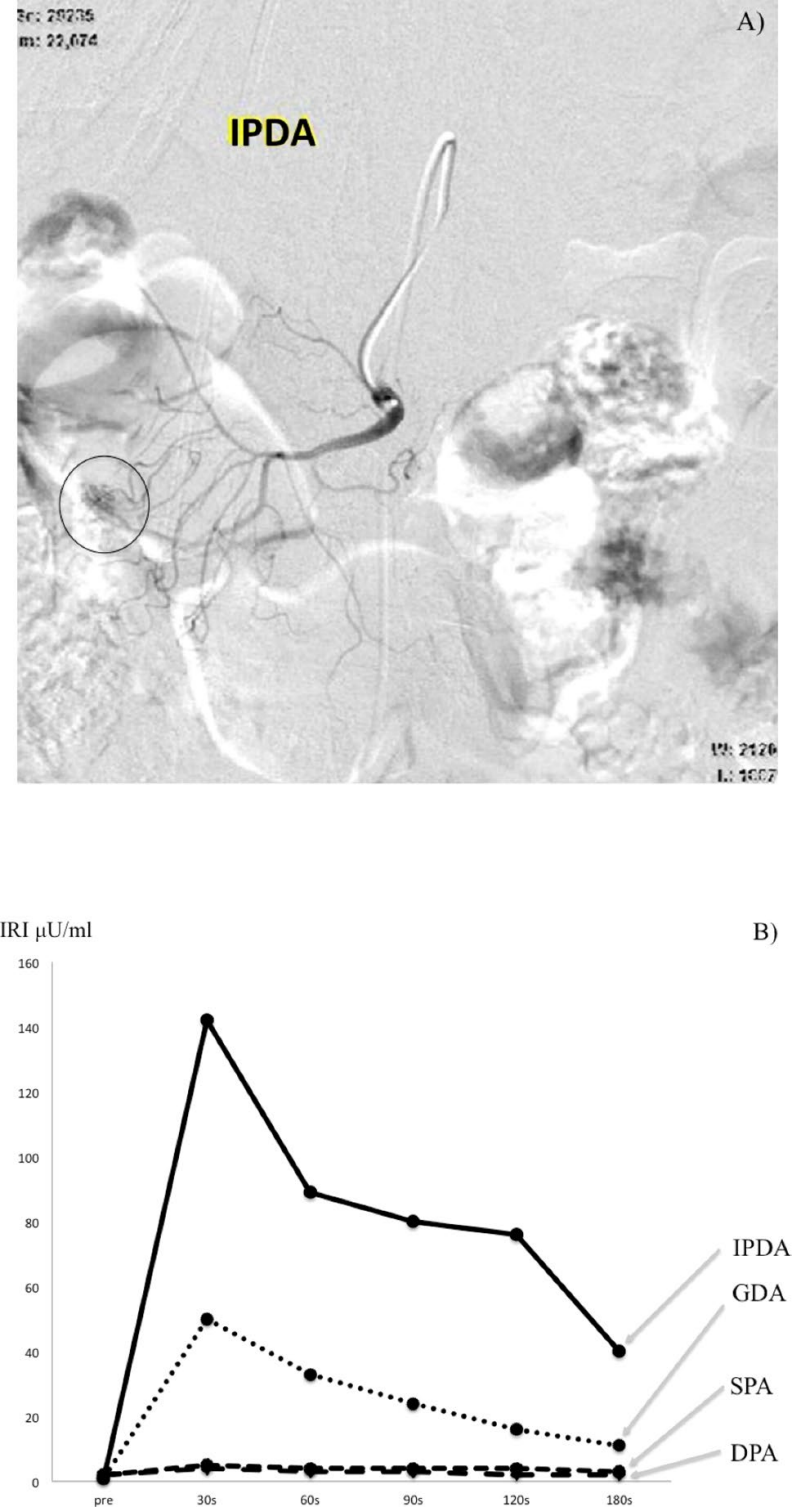
Results of the selective arterial calcium injection test in a patient with insulinoma. In this patient, immunoreactive insulin (IRI) levels increased at 30 s after the injection into the inferior pancreaticoduodenal artery (IPDA) (**A**, **B**). *GDA* gastroduodenal artery; *SPA* splenic artery, *DPA* distal pancreatic artery.

Before angiography, a 5-Fr shepherd hook catheter (Hanako Medical, Terumo, Japan) was placed in the right or middle hepatic vein through the right femoral vein. In addition, a 5-Fr catheter was placed in the celiac artery and superior mesenteric artery [SMA] through the right femoral artery followed by insertion of a microcatheter (Progreat ∑, Terumo, Japan) into an artery feeding the pancreas (e.g., the common hepatic artery [CHA], gastroduodenal artery [GDA], splenic artery [SPA], distal pancreatic artery [DPA], or inferior pancreaticoduodenal artery [IPDA]). Calcium gluconate at a dose of 0.025 mEq/kg was diluted to a 4 mL bolus, and injected rapidly into each artery. Blood samples for insulin or gastrin were obtained from the right hepatic vein at 30, 60, 90, 120, and 150 or 180 s after the calcium injection. The results were considered positive for an insulinoma if the maximum increase in serum levels of immunoreactive insulin (IRI) was twice at 30–60 s compared to the basal serum IRI level^2^. In contrast, for gastrinomas, the results were considered positive when serum levels of immunoreactive gastrin (IRG) were > 80 pg/ml at 40 s after the calcium injection^9^, and IRG was > 20% above the basal serum IRG at 40 s after the calcium injection^9^. When a positive response was observed in the SPA and DPA, the tumor was considered to be localized to the body or tail of the pancreas; when it was identified in the CHA, SMA, GDA, and IPDA, the tumor was considered localized to the pancreatic head (Fig. 1).

### Surgical resection and pathology

Surgical procedures included enucleation, partial pancreatectomy, laparoscopic or open distal pancreatectomy, and pancreaticoduodenectomy. Pathological staging was performed according to the Union for International Cancer Control Tumor-Node-Metastasis (UICC-TNM) Classification of Malignant Tumors (8^th^ edition), and tumors were classified as PanNET G1, PanNET G2, PanNET G3, and PanNEC (G3) according to the 4^th^ edition of the WHO Classification of Tumors of Endocrine Organs. Immunostaining was performed for chromogranin A, synaptophysin, CD56, insulin, gastrin, somatostatin, glucagon, and pancreatic polypeptide. Moreover, pancreatic neuroendocrine microadenomas, which are benign non-functioning neuroendocrine tumors < 0.5 cm with a low proliferation rate, were also counted.

### Postoperative assessment and follow-up

We evaluated the postoperative symptoms and the IRI or IRG levels, and all the patients were followed-up at 1, 3, 6, and 12 months after surgery. Patients also underwent semiannual reviews. In addition, clinical examinations, laboratory investigations, and abdominal computed tomography (to detect tumor recurrence) were performed.

### Informed consent statement

Patients were not required to give informed consent to the study because the analysis used anonymous clinical data that were obtained after each patient agreed to treatment by written consent. This statement was approved by the Human Experimentation Committee of Keio University School of Medicine.

## Results

### Demographic and perioperative characteristics

A total of 14 patients underwent the SACI test and curative resection for F-PNETs between January 1997 and September 2016. The patient characteristics are summarized in Table 1. There were 7 men and 7 women, with a median age of 55 years (range, 18–75 years). The most common clinical presentations were hypoglycemia (n = 11, 78.6%), followed by melena (n = 1, 7.1%) and elevation in glycated hemoglobin levels (n = 1, 7.1%); 1 patient (7.1%) was asymptomatic. The diagnosis was insulinoma in 11 patients (78.6%) and gastrinoma in 3 (21.4%). Five patients (35.7%) underwent open or laparoscopic distal pancreatectomy, 4 (35.7%) underwent partial pancreatectomy, 4 (35.7%) underwent open or laparoscopic enucleation, and 1 (7.1%) underwent pancreaticoduodenectomy. Three patients (no. 3, 4, and 7; 21.4%) were diagnosed with multiple endocrine neoplasia type 1 (MEN-I). After surgery, all patients showed no symptoms, and the levels of IRI and IRG improved. Patient no. 4 had bone metastasis as well as metastases in the lymph nodes of the aorta; the patient died approximately 5 years after surgery.

**Table 1:** Clinical and biochemical characteristics of the study patients (N = 14).

No	Age (y)	Gender	Preoperative symptom	Diagnosis	Operation	Hereditary disease	Post operative symptoms	Pre operative serum insulin (μU/ml)	Post operative serum insulin (μU/ml)	Preoperative serum gastrin (pg/ml)	Post operative serum gastrin (pg/ml)	OS (months)	Recurrence	Status
1	36	M	Hypoglycemia	Insulinoma	PP	–	N	149	8	–	–	254	None	Alive
2	51	F	Hypoglycemia	Insulinoma	DP	–	N	51	2	–	–	233	None	Alive
3	70	M	Hypoglycemia	Insulinoma	EN	MEN–I	N	164	6	–	–	119	None	Alive
4	38	M	Hypercalcemia	Insulinoma	DP	MEN-I	N	34	8	–	–	59	Bone, lymph node (Ao)	Dead
5	58	M	Melena	Gastrinoma	PP	–	N	–	–	17,800	39	87	None	Alive
6	48	F	Hypoglycemia	Insulinoma	PP	–	N	272	2	–	–	85	None	Alive
7	43	M	Asymptomatic	Gastrinoma	PP	MEN-I	–	–	–	911	50	80	None	Alive
8	67	F	Hypoglycemia	Insulinoma	EN (Lap)	–	N	44	4	–	–	66	None	Alive
9	18	M	Hypoglycemia	Insulinoma	DP (Lap)	–	N	67	11	–	–	60	None	Alive
10	74	F	Hypoglycemia	Insulinoma	PD	–	N	142	10	–	–	45	None	Alive
11	33	F	Hypoglycemia	Insulinoma	EN (Lap)	–	N	50	4	–	–	31	None	Alive
12	75	M	Hypoglycemia	Insulinoma	DP (Lap)	–	N	39	4	–	–	30	None	Alive
13	58	F	Hypoglycemia	Insulinoma	EN	–	N	10	3	–	–	28	None	Alive
14	70	F	Elevation of HbA1c	Gastrinoma	DP (Lap)	–	–	–	–	994	387	24	None	Alive

*Ao* aorta, *DP* distal pancreatectomy, *EN* enucleation, *Lap* laparoscopic, *MEN-I* multiple endocrine neoplasia type 1, *N* none, *OS* overall survival, *PD* pancreaticoduodenectomy, *PP* partial pancreatectomy.

The results of preoperative imaging studies in 14 patients (dynamic CT, MRI, CTA, and the SACI test) are shown in Table 2. The detection rate of F-PNET was 78.6% for CT, 91.7% for MRI, 100% for CT and MRI, and 100% for CTA. We classified these patients into groups A, B, and C; group A, one tumor detected by either CT or MRI; group B, multiple tumors detected; and group C, the tumor location was accordant on CT, MRI, and CTA, but the SACI test revealed another tumor. In group A consisting of patient no. 1, 2, 5, 6, 8, 9, 10, 11, 12, and 13, the tumor was also detected by CTA and the SACI test was positive on calcium injection. In group B consisting of patient no. 3, 4, and 7, the focus tumor among the multiple tumors was detected by the SACI test. Moreover, all patients in group B had MEN-I. In group C consisting of patient no. 14, another tumor was identified by the SACI test, whose location was different from that detected using CT, MRI, and CTA.

**Table 2:** Results of preoperative imaging studies (N = 14).

No	Group	SACI test		CT			
		Feeding artery	Location	Number	Location (number)	Size (mm)	Findings
1	A	GDA	H	1	H	20	Hypo
2	A	SPA	T	1	T	40	Hyper
3	B	CHA	H	1	B	12	Hyper
4	B	IPDA	H	3	H(1), B (2)	30(B), 15(H), 5(B)	Hyper
5	A	SMA, IPDA	H	1	H	30	Hyper
6	A	GDA	H	1	H	12	Hyper
7	B	GDA, IPDA	H		ND		
8	A	SPA	T	1	T	14	Hyper
9	A	SPA	T	1	T	35	Hyper
10	A	GDA, IPDA	H	1	H	25	Hyper
11	A	GDA, IPDA	H	1	H	10	Hyper
12	A	IPDA	B		ND		
13	A	GDA	H		ND		
14	C	GDA, IPDA	H	1	B	9	Hyper

No	MRI				CT Angiography		
	Number	Location (number)	Size (mm)	Findings	Number	Location (number)	Size (mm)
1	1	H	20	T1 low, T2 high	1	H	20
2	1	T	40	T1 low, T2 high	1	T	40
3	2	H, B	12(B), 6(H)	T1 low, T2 high, hyper	2	H, B	H(10), B(14)
4	3	H(1), B(2)	30(B), 15(H), 5(B)	T1 low, T2 high, hyper	6	H(3), B(2), T(1)	30(B), 8(B), 5(H), 4(H), 2(B), 2(T)
5	1	H	35	T1 low, T2 high, hyper	1	H	30
6		ND			1	H	15
7		NP			2	H	10(H), 7(H)
8	1	T	12	T1 low, T2 low, hyper	1	T	15
9	1	T	35	T1 low, T2 low	1	T	30
10	1	H	18	T1 low, T2 iso, hyper	1	H	20
11		NP			1	H	10
12	1	B	10	T1 low, T2 high, hyper		ND	
13	1	H	9	T1 low, T2 iso, hyper	1	H	10
14	1	B	9	T1 low, T2 high	1	B	7

*B* body, *CT* computed tomography, *GDA* gastroduodenal artery, *H* head; hyper: hypervascular, *hypo* hypovascular, *IPDA* inferior pancreatoduodenal artery, *MRI* magnetic resonance imaging, *ND* not detected, *NP* not performed, *SACI* selective arterial calcium injection, *SMA* superior mesenteric artery, *SPA* splenic artery, *T* tail.

### Pathological findings

The pathological findings of the tumors are shown in Table 3. The UICC stage was 1A in 10 patients (71.4%) and 1B in 4 (28.6%), and PanNET G1 was detected in 9 patients (64.3%) and PanNET G2 in 5 (35.7%). Patients in group B had a tendency of having pancreatic neuroendocrine microadenomas.

**Table 3:** Pathological findings (N = 14).

No	Group	UICC-TNM stage*	NET	Immunostaining								Findings		
			G1/G2/NEC	chro A	CD56	Syn	Insulin	Gastrin	Soma	Glu	PPP	Number	Size (mm)	Microadenoma
1	A	1B	G2	p	–	–	p	–	p	–	–	1	15	0
2	A	1B	G1	p	–	–	pp	–	n	–	–	1	30	0
3	B	1A	G2	p	–	p	p	n	n	n	–	2	12, 8	0
4	B	1B	G1	n	–	p	p	n	n	n	n	21	0.4–26	15
5	A	1A	G2	p	–	p	n	p	pp	n	n	1	30	0
6	A	1A	G2	p	pp	p	p	n	p	n	n	1	8	0
7	B	1A	G2	p	–	p	n	p	n	n	n	4	0.4–8	3
8	A	1A	G1	p	p	p	p	n	–	n	–	1	13	0
9	A	1B	G1	p	–	p	p	n	n	n	n	2	30, 2	1
10	A	1A	G1	p	–	p	p	n	n	n	n	1	16	0
11	A	1A	G1	p	p	p	p	n	n	n	n	1	8	0
12	A	1A	G1	p	p	p	p	n	pp	n	–	1	14	0
13	A	1A	G1	p	p	p	p	n	n	n	–	1	8	0
14	C	1A	G1	p	pp	p	n	n	n	n	n	1	7	0

*chro A* chromogranin A, *Glu* glucagon, *n* negative, *PPP* pancreatic polypeptide, *pp* partial positive, *p* positive, *Syn* synaptophysin, *Soma* somatostatin, *UICC-TNM* Union for International Cancer Control Tumor-Node-Metastasis.

*Pathological stage, UICC TNM Classification of Malignant Tumors 8th edition.

## Discussion

The study findings demonstrated that the SACI test should be performed when multiple tumors are detected on CT or MRI with inheritable characteristics such as MEN-I; this will help in localizing the causative tumor among multiple tumors as well as in deciding the indications for the extent of surgery to resect the tumors in the pancreas. However, the SACI test or CTA should not be performed if a single tumor is detected via CT and MRI because the SACI test is relatively invasive compared to CT or MRI. Nevertheless, we need to be careful about false-positive results or occult tumors, such as those observed in no. 14 in which the tumor location revealed on the SACI test differed from that detected on CT and MRI.

According to international guidelines such as the European Neuroendocrine Tumor Society (ENETS)^10^, the North American Neuroendocrine Tumor Society (NANETS)^11^ and the National Comprehensive Cancer Network (NCCN)^12^, the SACI tests should be considered in patients with F-PNETs when other imaging tests, such as CT or MRI, are equivocal or negative. Furthermore, most experts recommend this test only for patients with persistent or recurrent insulin-mediated hypoglycemia. According to the ENETS guideline^10^, the SACI tests should be performed in patients with multiple lesions or MEN-I, and excision should be performed based on the results of the SACI tests. Our results were consistent with the ENETS guideline, but not other guidelines. Therefore, this study supported the adaptation of the SACI tests mentioned in the ENETS consensus guidelines.

The SACI test is a method for the identification and differentiation of insulinomas or gastrinomas among multiple NETs that are essentially impossible to identify on imaging techniques alone, and it is useful for planning the surgical strategy preoperatively^3–5^. In patient no. 3 and 7 in group B, the SACI test revealed accurate localization among multiple NETs, and we performed the surgical procedure according to the result of the SACI test. However, in no. 4 in group B, although the SACI test revealed the tumor in the pancreatic head, we performed distal pancreatectomy and not pancreaticoduodenectomy. Although we planned pancreaticoduodenectomy and enucleation for the tumor in the pancreatic body in no. 4 preoperatively, there was a main pancreatic duct injury while performing enucleation. We could not perform total pancreatectomy per the patient’s wishes, so we had no choice but to perform distal pancreatectomy. However, fortunately, the symptoms of hypercalcemia and postoperative IRI improved after surgery; therefore, we suspected that the tumor in patient no. 4 was in the pancreatic body, and that the SACI test did not always reveal the correct localization. In fact, Doppman et al.^2^ found that elevated hepatic venous insulin levels were not always correlated with the correct localization: unequivocal localization was reported in only 5 of 9 patients (56%) but a gradient in venous insulin levels was observed in all 9 patients.

EUS plays a specific role in PNETs, but the necessity of EUS is a little different according to the international guidelines from ENETS^10^, NANETS^11^, and NCCN^12^. These guidelines recommended that EUS can be used as appropriate if there are equivocal CT or MRI findings, or if the tumors were not detected by them. Furthermore, EUS is recommended if pancreatic resection is considered for assessing and localizing tumors preoperatively. The NANETS guideline^11^ suggested that EUS should be performed when there is a question of multifocality, such as in patients with MEN1, and EUS can be useful when some F-PNETs are too small to be imaged with CT or MRI. EUS-FNA can also be considered to confirm the diagnosis and tumor grade, although tumor heterogeneity may preclude accurate assessment; thus, EUS or EUS-FNA can also be considered as additional imaging tools instead of CT or MRI in the above specific situations^11^.

Recently various studies^10–14^ have reported that positron emission tomography with CT (PET/CT) with ^68^ Ga-labeled somatostatin analogues (^68^ Ga-PET/CT) has the highest sensitivity for localizing not also NF-PNETs but also FP-NETs, and is also a cornerstone tool for both diagnosis and staging for PNETs. The sensitivity varies from 86–100%, and the specificity from 79–100% for all p-NETs. Thus, the SACI test, which is relatively invasive, could be omitted in patients with F-PNETs, especially those with gastrinomas. Furthermore, international guidelines^10–12^ have recommended ^68^ Ga-PET/CT as the first-line diagnostic imaging method for staging in patients with gastrinoma. However, ^68^ Ga-PET/CT is not useful for insulinoma. Shorma P et al. revealed that its sensitivity is only 25%^11^. Although the adaptation and usefulness of the SACI test has been limited because other non-invasive functional imaging techniques, such as ^68^ Ga-PET/CT, are being used instead to determine surgical planning, staging, and medical management, the SACI tests for insulinoma may still useful under special conditions for patients with FP-NETs (especially insulinoma) with multiple lesions or patients with MEN-I where CT or MRI are equivocal or negative.

If F-PNETs are not detected on preoperative imaging studies, i.e. if they are “occult tumors,” blind distal, subtotal, or total pancreatectomy is now obsolete. These blind pancreatic resections are no longer indicated and should not be performed because of their short-term (e.g., pancreatitis, pancreatic fistula, and pancreatic abscess) and long-term morbidities (e.g., exocrine pancreatic insufficiency and diabetes mellitus)^6,15^. Indications for occult insulinomas and gastrinomas have been reported in ENTES or NCCN guidelines^10,12^. First, for occult insulinoma, observation and surgical exploration are options for occult tumors. Few studies have advocate intraoperative localization through palpation, and the addition of intraoperative ultrasound may be more sensitive than invasive preoperative methods^1,16,17^. Second, for gastrinomas, exploratory surgery including duodenotomy or local resection/enucleation of tumors, periduodenal node dissection, and intraoperative ultrasound have been considered in a previous report^12^. In patient no. 14 in group C, the tumor location revealed on the SACI test was different from the location observed on CT, MRI, and CTA. Based on the result of the SACI test, the tumor might have been located in the pancreatic head although the tumor was not detected on CT, MRI, and CTA; therefore, the tumor might have been an occult tumor. Another alternative could be that the result of the SACI test might have been a false-positive result and that there was no tumor there. After we informed the patient of these two possibilities, we performed distal pancreatectomy, instead of blind pancreaticoduodenectomy, considering the postoperative surgical complications. Although we performed intraoperative ultrasonography for the pancreatic head, a tumor was not detected. In addition, during the semiannual clinical examinations, laboratory investigations, and abdominal computed tomography, a new tumor was not detected in the remnant pancreas and the IRG level decrease to 170 pg/ml. Therefore, in this case, we suspect that the result of the SACI test might be a false-positive result and that there was no tumor at that location. According to the NCCN guidelines^12^, we may consider exploratory surgery including intraoperative ultrasound in the future for patient #14 in our study if the patient’s IRG level continue to increase.

We suggest that the SACI test and CTA do not need to be performed if a single tumor is detected on CT and MRI. The preoperative detection rates of CT and MRI for PNETs depended on the patient age and type of study^7,18,19^, although the development in the technology of scanners has resulted in a marked improvement in the sensitivity of CT and MRI^6,7^. In this study, the detection rate of F-PNETs was 100% for a combination of dynamic CT and MRI, and if a single tumor was observed, the SACI test was not necessary for checking the localization of the single tumor to administer the calcium injection because we should not perform blind pancreatectomy for occult PNETs. CTA, an invasive localization modality, is considered the “gold standard” for tumor detection, and the detection rates varied from 29 to 100% depending on the experience of the operator and the center^6,9,20,21^. In our institution, CTA has often been performed for not only F-PNETs but also NF-PNETs, and as shown in Table 2, CTA may be useful to detect multiple small tumors with hereditary disease, such as MEN-I.

This study has a few limitations. First, this was a retrospective study conducted at a single institution with a relatively small number of patients. However, as the number of patients with F-PNETs is small and most previous studies were case series^22–24^, a multi-center study with many patients is needed to further evaluate the usefulness of the SACI test. Second, the time span of this study (from 1997 and 2016) is very long considering the technical improvements of both CT and MRI. In this study, two patients underwent surgery in 1997 and 1999, and 12 patients had undergone pancreatic surgery since 2007, in which multi-detector row CT or dynamic MRI had a mechanical efficacy similar to the recent CT or MRI technology. Although we considered excluding these two patients, there are only a few patients that undergo the SACI tests and this study already had a small number of patients; thus, we included these two patients and the time span became long. Finally, among 14 patients, 8 patients (57.1%) underwent EUS^10–12,25^, 4 (28.6%) underwent 18F-FDG PET/CT^26^, and 2 (14.3%) underwent somatostatin receptor scintigraphy (SRS)^27^. We have been performing EUS on patients with pancreatic neuroendocrine tumors (PNETs) since 2007, and now we perform EUS more actively in most patients with PNETs, future prospective research studies are needed to confirm and evaluate these preliminary findings, by including EUS, 18F-FDG PET/CT, and ^68^ Ga-PET/CT, all of which are useful to evaluate the location of PNETs.

In conclusion, the SACI test should be performed for F-PNETS with multiple tumors detected on CT or MRI, especially for those with inheritable characteristics such as MEN-I. This will help in determining the tumor localization among multiple tumors and deciding the appropriate surgical method for the treatment of the multiple tumors in the pancreas. Moreover, the SACI test or CTA should not be performed if a single tumor is detected via CT and MRI, because we should not perform blind pancreatectomy against occult F-PNETs.
